# Heterogeneous Fenton-Like Catalysis of Electrogenerated H_2_O_2_ for Dissolved RDX Removal

**DOI:** 10.3389/fceng.2022.864816

**Published:** 2022-05-03

**Authors:** Patrick Compton, Nazli Rafei Dehkordi, Michael Knapp, Loretta A. Fernandez, Akram N. Alshawabkeh, Philip Larese-Casanova

**Affiliations:** Department of Civil and Environmental Engineering, Northeastern University, Boston, MA, United States

**Keywords:** Explosives, oxidation processes, heterogenous catalysts, electroperoxidation, RDX

## Abstract

New insensitive high explosives pose great challenges to conventional explosives manufacturing wastewater treatment processes and require advanced methods to effectively and efficiently mineralize these recalcitrant pollutants. Oxidation processes that utilize the fundamental techniques of Fenton chemistry optimized to overcome conventional limitations are vital to provide efficient degradation of these pollutants while maintaining cost-effectiveness and scalability. In this manner, utilizing heterogeneous catalysts and *in-situ* generated H_2_O_2_ to degrade IHEs is proposed. For heterogeneous catalyst optimization, varying the surface chemistry of activated carbon for use as a catalyst removes precipitation complications associated with iron species in Fenton chemistry while including removal by adsorption. Activated carbon impregnated with 5% MnO_2_ in the presence of H_2_O_2_ realized a high concentration of hydroxyl radical formation - 140 μM with 10 mM H_2_O_2_ - while maintaining low cost and relative ease of synthesis. This AC-Mn5 catalyst performed effectively over a wide pH range and in the presence of varying H_2_O_2_ concentrations with a sufficient effective lifetime. *In-situ* generation of H_2_O_2_ removes the logistical and economic constraints associated with external H_2_O_2_, with hydrophobic carbon electrodes utilizing generated gaseous O_2_ for 2-electron oxygen reduction reactions. In a novel flow-through reactor, gaseous O_2_ is generated on a titanium/mixed metal oxide anode with subsequent H_2_O_2_ electrogeneration on a hydrophobic microporous-layered carbon cloth cathode. This reactor is able to electrogenerate 2 mM H_2_O_2_ at an optimized current intensity of 150 mA and over a wide range of flow rates, influent pH values, and through multiple iterations. Coupling these two optimization methods realizes the production of highly oxidative hydroxyl radicals by Fenton-like catalysis of electrogenerated H_2_O_2_ on the surface of an MnO_2_-impregnated activated carbon catalyst. This method incorporates electrochemically induced oxidation of munitions in addition to removal by adsorption while maintaining cost-effectiveness and scalability. It is anticipated this platform holds great promise to eliminate analogous contaminants.

## INTRODUCTION

New insensitive high explosives (IHE)–fire/shock resistant components of insensitive munitions–as well as traditional munitions constituents (MC) pose challenges to conventional treatment of explosive manufacturing waste streams and areas of groundwater contamination. Innovative approaches that maintain cost-effectiveness of large-scale treatments are vital to ensure safety for human health and the environment. Both legacy MCs such as trinitrotoluene (TNT), hexahydro-1,3,5-trinitro-1,3,5-triazine (RDX), and IHEs like 2,4-dinitroanisole (DNAN) and nitro-1,2,4-triazole-5-one (NTO) pose threats to human health and ecosystems when present in the environment (Warner et al., 2012; Dodard et al., 2013; Bannon and Williams, 2015; Johnson and Reddy, 2015; Olivares et al., 2016; Madeira et al., 2018). Various chemical, physical, and biological approaches for treatment of wastewater loaded with explosive compounds have been investigated in the past decades, yet it has been identified that the next generation IHEs provide new challenges that require innovative solutions beyond conventional treatment methods. While there are many options for treating these munitions constituents, emerging advanced oxidation processes (AOPs) are viable remediation techniques to degrade these recalcitrant pollutants. Almost entirely impervious to generally applied physicochemical water treatment processes, recalcitrant pollutants such as these IHEs are best suited for non-selective targeting treatment techniques (Krishnan et al., 2017). Due to the nature of these munitions’ constituents, they are susceptible to both reduction and oxidation methods for degradation.

Hydroxyl radicals generated via the Fenton process are strong oxidants able to non-selectively degrade surrounding compounds, making this AOP more advantageous than tailored chemical or biological treatment (Fenton, 1894). While the traditional Fenton process is constrained due to pH sensitivity, logistical concerns of utilizing external H_2_O_2_, and scalability, burgeoning single and coupled optimization techniques allow this process to be more viable for large-scale treatment implementation (Zhang et al., 2019). The photo-Fenton single optimization technique utilizes light irradiation to aid in the reduction of Fe(III) back to Fe(II) for use in the traditional homogenous Fenton process and makes the use of this iron catalyst more economic (Zhang et al., 2019). Heterogenous Fenton utilizes alternative catalysts than the traditional Fe(II) metal and allows for a wider pH range operability as well as reducing the production of iron sludge. Additionally, the electroperoxidation aspect of the electro-Fenton process generates H_2_O_2_ *in-situ* by electrochemically reducing oxygen on a cathode which is then reduced by a catalyst to produce hydroxyl radicals (Gao et al., 2015; Jiang et al., 2018).

Heterogeneous Fenton optimization is advantageous in providing a wider pH range for operations and creating more stable catalysts, primarily by impregnating or immobilizing iron species on durable supports. These can take the form of iron oxides impregnated on graphene supports (Guo et al., 2013; Liu et al., 2017), bimetallic catalysts on silica support structures (Karthikeyan et al., 2016), and clay plates coated in immobile iron species (Bel Hadjltaief et al., 2014). While these are highly stable and reusable catalysts, they are expensive and limited for large-scale applications in their present form (Nannan Wang et al., 2016; Ma et al., 2018). Granular activated carbon (GAC) is an inexpensive, stable, and manipulable support used in a range of engineering applications, to include for heterogenous Fenton optimization (Mirzaei et al., 2017). As a manipulable support in this capacity, GAC’s surface chemistry can be altered and utilized as a stable support structure for use in engineering applications (Qin et al., 2018). Indeed, this support has been utilized previously as a support for Fenton and Fenton-like catalysis (Saroyan et al., 2019), which utilizes catalysts other than iron to produce hydroxyl radicals in the presence of hydrogen peroxide. One family of Fenton-like catalysts, manganese oxides, is able to form abundant reactive oxygen species such as hydroxyl radicals after catalyzing the decomposition of H_2_O_2_ and subsequent chain reactions with free radicals (Kanungo et al., 1981). The mechanisms for the catalysis of H_2_O_2_ by manganese oxide compounds is similar to traditional Fenton processes, with the reduction of Mn(IV) to Mn(III) and reduction of H_2_O_2_ to free radicals–most notably hydroxyl radicals–which then can oxidize Mn(III) to Mn(IV) and complete the redox cycle (Wenmei Wang et al., 2016). This Fenton-like catalyst impregnated on GAC supports has been utilized previously (Saroyan et al., 2019) with promising results for hydroxyl radical production and subsequent pollutant degradation. As well, it removes the complications associated with iron catalysts such as iron leaching and precipitation in basic pH environments.

While certain single optimization techniques to the Fenton process - heterogeneous and photo-Fenton optimizations being the most prominent - utilize external addition of hydrogen peroxide, the transportation, handling, storage, and addition of external hydrogen peroxide into these systems is expensive and a great logistical burden. Electro-Fenton as an optimization process covers this constraint by generating hydrogen peroxide *in-situ* by electrochemical reduction of O_2_ on a cathode (Brillas and Martínez-Huitle, 2015). In addition to the reduction of O_2_, the cathode in an electro-Fenton system can act as a site to reduce Fe(III) generated by Fenton’s reaction back to Fe(II) and thus minimize initial iron salts concentration and iron sludge production (Ting et al., 2008). The electro-Fenton optimization process has the advantages of *in-situ* hydrogen peroxide generation and low iron sludge production but has to contend with a low hydrogen peroxide yield and low throughput of contaminants. Much research in the electro-Fenton realm has focused on optimizing operational parameters and cathode materials to enhance hydrogen peroxide electro-generation in cost-effective and environmentally friendly ways to circumvent this disadvantage (Khataee et al., 2011; Sheng et al., 2014; Yu et al., 2015). While not the only material that can cathodically reduce O_2_ to H_2_O_2_, carbon is the most utilized due to its chemical resilience and lower activity for further H_2_O_2_ decomposition. However, traditional carbon materials are less utilized than three-dimensional cathodes, such as graphite felts and active carbon fibers that have higher specific surface areas, current efficiencies, and reduction capabilities (Chaudhuri and Lovley, 2003; Panizza and Oturan, 2011; Wang et al., 2015; Castañeda et al., 2017).

While these single optimization methods are the fundamental first-order improvements made to essential Fenton chemistry, a multitude of researchers utilize coupled optimization processes which combine at least more than one of the above-mentioned single optimization methods in their attempts to treat various types of aqueous recalcitrant pollutants (Ifelebuegu and Ezenwa, 2011; El-Ghenymy et al., 2012; Sánchez Pérez et al., 2013; Babuponnusami and Muthukumar, 2014; Ganiyu et al., 2018; Wang et al., 2019). This coupling of optimization techniques is vital to the oxidation of legacy MCs and IHEs, which are resilient to generally applied physicochemical water treatment processes. Fenton and Fenton-like generation of hydroxyl radicals is well suited for this aim due to the ability of creating oxidants that are non-selective and combining optimization techniques to more efficiently and economically degrade these recalcitrant pollutants. Recent reviews on heterogeneous electro-Fenton and combined treatment options are focused on utilizing green technology for the removal of pharmaceuticals, catalytic mechanisms of various materials other than iron salts, and the process involved in hybrid and sequential processes for real wastewater treatment. The use of different materials as electro-Fenton catalysts (i.e., heterogeneous catalysts) is a growing trend due to pH regime operating limitations, though the main challenges involve performance enhancement, long-term operability, stability in various waste streams, cost-effectiveness, and overall life-cycle analysis (Meijide et al., 2021). Additionally, recent trends in research are focused on improving the catalytic activity of single atom catalysts for the degradation of organic pollutants, improving the yield of H_2_O_2_ production in electron-Fenton processes with more practical and highly efficient designs, and cost savings measures of stable catalysts that don’t require recapture from effluent waste streams (Wang et al., 2021). Finally, a review of many varying optimized Fenton process on specifically degrading insecticides reveals the need for robust, effective, energy-efficient, and non-selective targeting AOPs focused on the wide array of recalcitrant organic contaminants present in the environment today (Brillas, 2022).

The aim of this research is the removal and degradation of these recalcitrant pollutants utilizing a cost-effective MnO_2_-impregnated activated carbon heterogeneous catalyst in the presence of electrochemically generated H_2_O_2_ to create reactive oxygen species (ROS) such as hydroxyl radicals to oxidize MCs and IHEs, overcoming limitations to traditional Fenton with a design that is resilient, robust, and maintainable. These steps involve the electroperoxidation aspect of the electro-Fenton technique, which utilizes a 2-electron oxygen reduction reaction to form H_2_O_2_, and the heterogeneous Fenton optimization technique of using catalytic materials other than traditional iron ions to induce Fenton-like catalysis of this electrogenerated H_2_O_2_. The approach involves the optimization of reactor parameters to maximize the electrogeneration of H_2_O_2_ accumulation in the bulk solution, measure the effectiveness of the MnO_2_-impregnated AC in not only generating hydroxyl radicals but also removing a model pollutant, and traditional MC, RDX by adsorption and degradation, and designing a series of flow-through reactors to effectively incorporate these two coupled Fenton optimization techniques for removal of an RDX waste stream.

## EXPERIMENTAL

### Electrogeneration of H_2_O_2_

All electrogeneration tests were conducted in a flow-through electrochemical reactor. The reactor is a simple and cost-effective construction of inert, non-toxic, and high-density polyethylene with the ability to sustain long-term operation. Unless otherwise stated, baseline hydrogen peroxide generation tests were conducted with a 5 mM Na_2_SO_4_ electrolyte influent (purchased from Fisher Scientific), carbon cloth with microporous layers cathode (CCMPL) (purchased from FuelCellStore), 2 ml/min flowrate, and 150 mA constant current. pH was adjusted with either NaOH or H_2_SO_4_ (purchased from Fisher Scientific). H_2_O_2_ quantification was performed with 3 ml of the electrolyte solution extracted from flow after the cathode mixed with 0.5 ml of a synthesized TiOSO_4_. The concentration of the mixture was then quantified in a Shimadzu UV-1800 UV Spectrophotometer at 405 nm according to DIN 38 409, part 15, DEV-18. In figures where error bars are shown, results were obtained in duplicate.

### Synthesis of Heterogenous Catalyst and Batch Experiments

The modified GAC catalyst involved impregnating washed −20+40 mesh activated carbon (purchased from Fisher Scientific) with manganese dioxide nanopowder (nominal particle size 50 nm, purchased from United States Research Nanomaterials, Inc.). First, 500 mg of this MnO_2_ nanopowder was placed in DI water and sonicated for 10 min. 9.5 g of washed AC was then added, and the mixture was vortexed rapidly for 10 min. This mixture was then sealed and rotated for at least 48 h, filtered, and then dried in an oven at 110 °C for 24 h. The resultant catalyst was GAC with 5% w/w MnO_2_ nanopowder to AC, dubbed AC-Mn5.

For batch tests, either 50 ml of 10 mM benzoic acid (purchased from Fisher Scientific) for hydroxyl radical quantification, or 25 mg/L RDX (synthesized in lab) as a model MC pollutant were utilized. The solutions were well-mixed, with external H_2_O_2_ (purchased from Fisher Scientific) and varying masses of AC-Mn5 placed in solution for experimentation. The quantification for hydroxyl radicals follows from the formation of 4-hydroxybenzoic acid after the oxidation of benzoic acid by hydroxyl radicals. The benzoic acid and hydroxylated isomer byproducts were quantified by high-performance liquid chromatography with a UV detector at 254 nm using an Agilent 1260 Infinity Quaternary LC with an eluent of 80% HPLC grade water adjusted with phosphoric acid to a pH ∼2 and 20% methanol. The RDX solutions were quantified by HPLC with an eluent of 50% HPLC grade water and 50% methanol.

### Analytical Techniques for Byproduct Determination

Organic degradation products of RDX were identified using LC-MS-MS. Quantitative measurements and non-target screening was performed using a QTrap 4500 (AB Sciex) mass spectrometer paired with a Shimadzu Prominence HPLC system. The curtain gas pressure was set to 20 psi, the IonSpray voltage was set to −4500 V, the source temperature was set to 360°C, GS1 (nebulizer gas pressure) was set to 60 psi, and the GS2 (auxiliary gas pressure) was set to 60 psi. Separation was performed with a Hypersil Gold PFP column (Thermo Scientific, 2.1mm x 100mm, 3 μm) using 10 mM ammonium formate in water and 10 mM ammonium formate in methanol as the mobile phases at a combined flow rate of 0.5 ml min^−1^. For targeted analysis a gradient was run from 35% methanol to 98% methanol over 6.8 min. For non-targeted analysis a gradient was run from 2% methanol to 98% methanol over 22 min. The first 2 min of chromatography eluent were wasted to avoid injection of the Na^+^ and SO_4_^2−^ salts into the MS.

Inorganic degradation products nitrite, nitrate, acetate, and formate were measured by ion chromatography (Dionex DX-120 with 9 mM Na_2_CO_3_ eluent at 1.5 ml min^−1^ through a AS9-HC column), and ammonium was measured using an ammonium probe (Orien High Performance ammonia Probe). A portable pH/ORP/Temperature meter (Orion Star A221) was used to measure pH. Possible leaching of Mn from the AC-Mn5 column during operation was checked by inductively coupled plasma–mass spectrometry (Bruker Aurora M90, serviced by Analytik Jena). Total Organic Carbon (TOC) measurements were measured by a Shimadzu TOC-L series TOC analyzer.

### Combined Reactors for H_2_O_2_ Electrogeneration and RDX Removal

**FIGURE 1 |:**
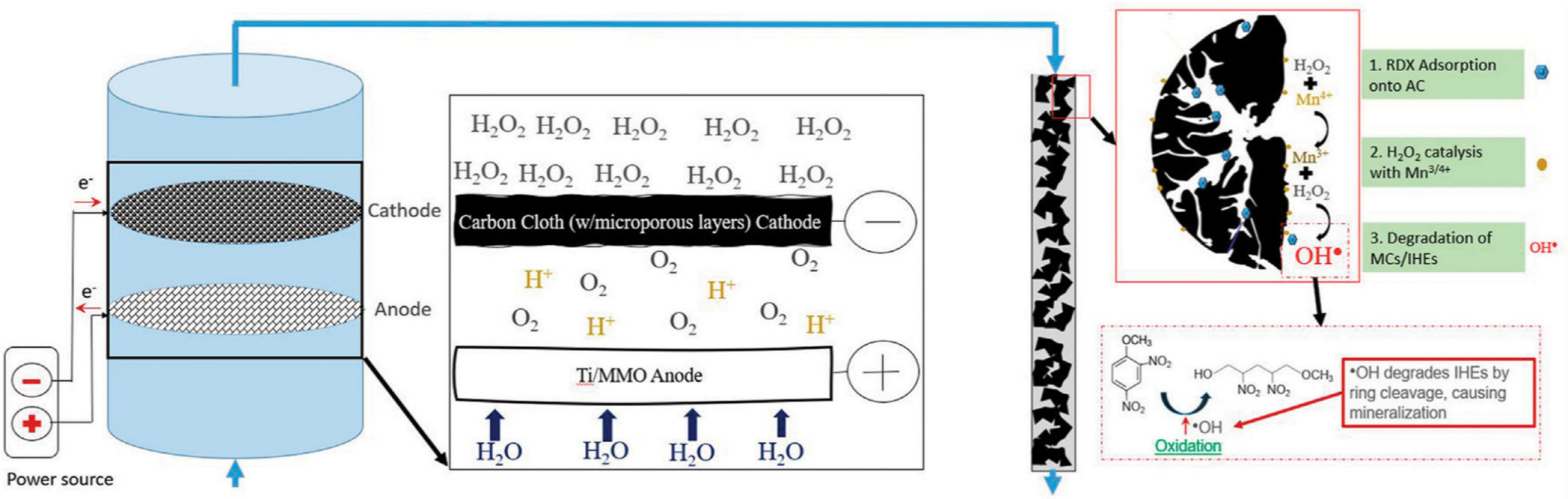
Setup for combined H_2_O_2_ generation in a plug flow reactor and subsequent model pollutant removal in packed AC-Mn5 column.

The setup for the final combined electroperoxidation and RDX removal by adsorption/Fenton-like catalysis experiments is shown in Figure 1. H_2_O_2_ electrogeneration in the plug flow reactor is described in Section 2.1, with a schematic representation shown in Figure 1. The lab-scale packed bed column utilized has a surface area of 2.63 cm^2^ and length of 2.5 cm. The bulk solution of the model pollutant RDX, electrolyte, and accumulated electrogenerated H_2_O_2_ is fed to the open-air end of the column to gas off any excess O_2_ filtered passed the CCMPL cathode. This mixture then percolates through the column where adsorption, catalysis, and degradation *via* hydroxyl radical oxidation occurs.

## RESULTS

### Cathode Material Selection

Carbon materials as cathodes are favorable over other efficacious metallic materials such as titanium, gold, or mercurial amalgams due to their current density range, cost, and relative availability (Petrucci et al., 2016). Materials that are commercially available with no chemical alterations before experimentation have the added benefit of ease of use and reactor processing time. Hydrophobic cathodes utilize gaseous O_2_ generated at an anode surface and have a higher surface area and pore volume for enhanced H_2_O_2_ electrogeneration. Supplementary Figure S1, provided in the supplemental, highlights this fact with the comparison of two hydrophilic cathodes–carbon felt and carbon cloth–and the hydrophobic CCMPL cathode to generate H_2_O_2_.

Due to the hydrophobic nature of the CCMPL cathode, H_2_O_2_ generation is nearly tenfold higher after 20 min of operation owing to the accumulation and slower diffusion of O_2_ bubbles over the hydrophilic cathodes. This mass generation rate increases rapidly at first over the first 30 min and diminishes slightly but still increases to nearly 46 mg/L (∼1.3 mM) after a 2-h runtime. While clearly evident that the carbon cloth with added microporous layers is much more advantageous at generating H_2_O_2_ *in-situ*, the hydrophobic nature of the carbon surface facing the O_2_ generating anode can lead to possible bubble oversaturation. Many systems that implement electro-Fenton techniques with carbonaceous cathode materials experience reduced efficacy from the mismanagement of gas bubble formation (Taqieddin et al., 2017). While oxygen bubbles accumulate and subsequently coalesce in the cathode adherence zone, H_2_O_2_ generation will continue to increase. However, there is a strong possibility that these bubbles will coalesce so far as to completely disassociate the cathode material from the waste stream, leading to a rapid drop in voltage and ensuing drop in H_2_O_2_ production. This system is no exception; however, the implementation of the flexible microporous layered carbon cloth allows for a wide range of size/orientation manipulation to allow for oxygen bypass. Supplementary Figure S2, provided in the supplemental, shows the size comparison and respective H_2_O_2_ production of a small diameter cathode with constant O_2_ bubble bypass, large diameter cathode with no bypass, and a large diameter cathode with a bypass to prevent oversaturation.

It is clear that while rapid accumulation of O_2_ bubbles is advantageous for H_2_O_2_ generation, oversaturation leads to a rapid decrease in generation once the coalesced O_2_ disassociates the cathode from the waste stream. Oversaturation occurs more quickly and causes cathode disassociation the higher the current applied in this system due the higher rate of O_2_ generation. As well, possible parasitic reactions such as an additional 4-electron oxygen reduction of H_2_O_2_ to water on the cathode surface may occur the higher current applied as electrogenerated H_2_O_2_ can disassociate on the same cathode surface.

### Parameter Optimization to Maximize H_2_O_2_ Electrogeneration

**FIGURE 2 |:**
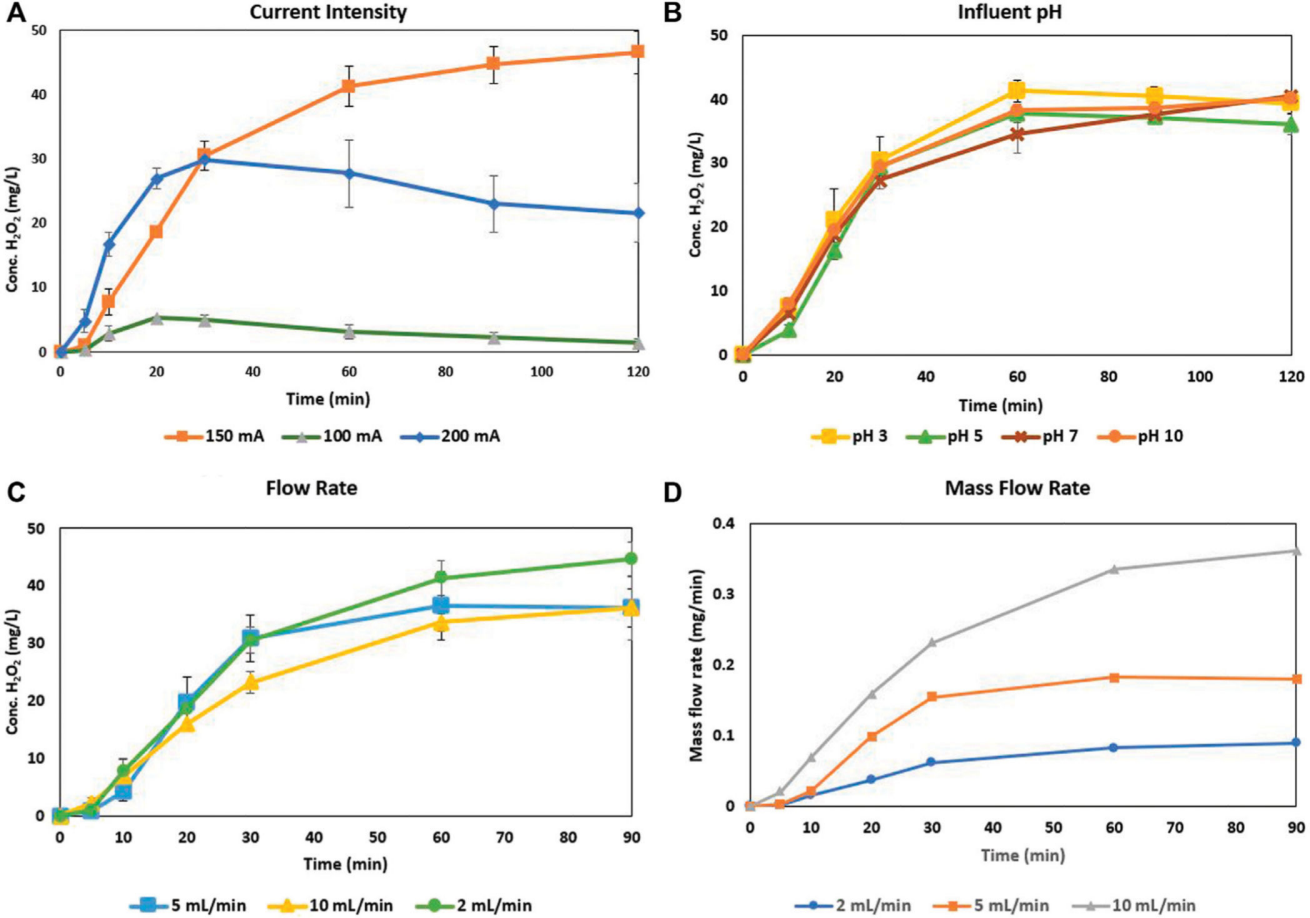
H_2_O_2_ electrogeneration optimization for **(A)** Current intensity **(B)** Influent pH values **(C)** Influent flow rate, and **(D)** H_2_O_2_ mass flow rate.

Figure 2 details the optimized current intensity based on these factors, as well as the concentration of H_2_O_2_ electrogenerated at varying flow rates and influent pH values. The current intensity is the driving variable in this reactor setup and is relative to the cathode/reactor diameter, as the intensities of 100, 150, and 200 mA correspond to densities of 6.3, 9.5, and 12.5 mA/cm^2^, respectively. While a current intensity of 200 mA initially causes the most rapid increase in H_2_O_2_ generation, it quickly peaks and starts to decrease after 30 min. The inclusion of the error bars shows that the subsequent generation at 200 mA after 30 min is the most inconsistent of the intensities tested. At 150 mA current intensity, the reactor was consistently and predictably able to outperform in H_2_O_2_ electrogeneration over the other intensities. While pH is a master variable in the traditional sense, this reactor and cathode configuration was able to self-regulate (Supplementary Figure S4 appearing in the supplemental) and perform consistently over a wide range of influent pH values. The flow rate of the reactor is vital in determining the throughput of a contaminant and how effective H_2_O_2_ generation is with less contact time at the cathode surface. While the flow rate itself shows that the concentration of H_2_O_2_ electrogenerated decreases with increased flow rate, the mass flow rate shows that in fact more mass of H_2_O_2_ is generated per minute at higher flow rates allowing for faster throughput and increased mass of H_2_O_2_ for use in catalysis.

### Cathode Longevity and Reusability

**FIGURE 3 |:**
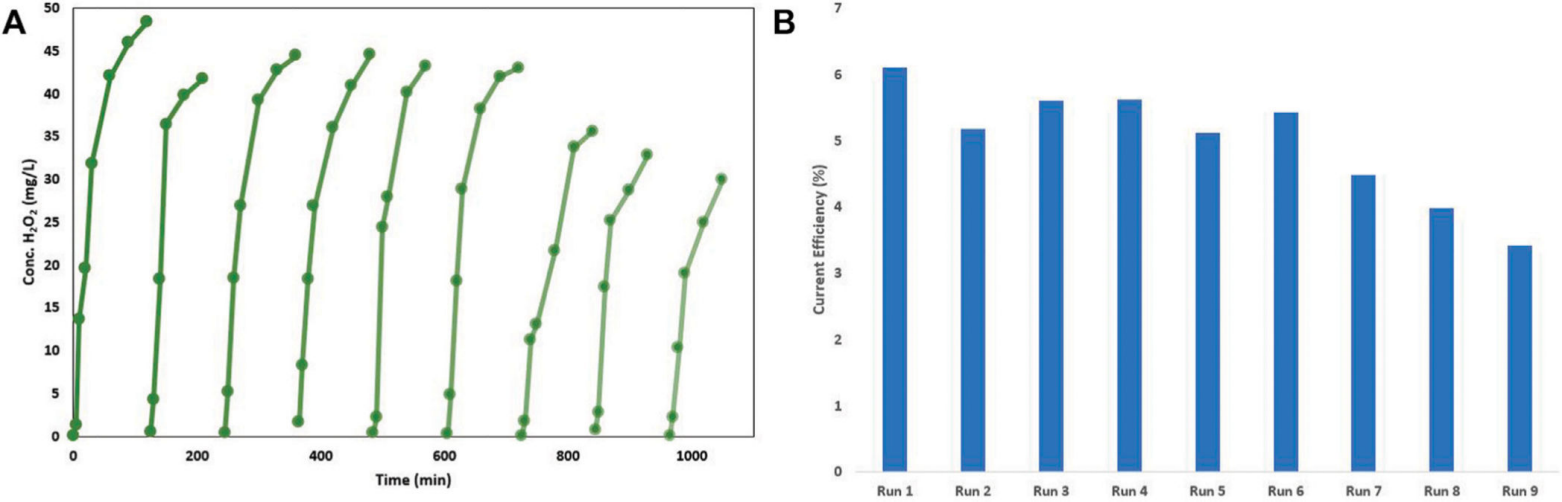
**(A)** Reusability of single CCMPL cathode for H_2_O_2_ electrogeneration and **(B)** corresponding steady state current efficiencies (CE) for each non-continuous consecutive run of H_2_O_2_ electrogeneration.

The longevity and reusability of the CCMPL cathode is vital in determining the maintenance and practicality of this system in industrial application. Figure 3A shows the results of nine consecutive H_2_O_2_ electrogeneration tests with the same CCMPL cathode. After nine non-continuous, consecutive runs with the sample CCMPL cathode, the material realized nearly 63% of the initial yield after >1000 min of operation. While it is evident that there is variability in the peak H_2_O_2_ concentration achieved–run two realized a concentration of 42 mg/L compared to 48 mg/L for run 1–there is no significant drop in efficacy until run 7 after over 12 h of operation. With this setup, the parameter that is most sensitive after optimal cathode orientation is established is the current density applied to the CCMPL cathode. In this vein, Figure 3B shows the current efficiency (CE) for H_2_O_2_ electrosynthesis for these nine non-continuous, consecutive trials. The values were determined *via* Eq. 1 (Wang et al., 2022),

(1) $$\mathrm{CE}=\frac{2\;x\;F\;x\;C_{H2O2}\;x\;V}{I\;x\;t}\times 100\%$$

where two is the number of electrons involved in the 2-electron ORR, F is the Faraday constant (96,485 Coulombs/mol), C_H2O2_ is the electrogenerated H_2_O_2_ concentration (M), V is the solution volume (L), I is the applied current (A), and t is the time of operation (seconds). The flow-through reactor setup utilizing this cathode is able to handle a wide-range of influent pH values, maintain effectiveness with increasing flow rate throughput, and continue to generate H_2_O_2_ *in-situ* over multiple consecutive operations. This is vital to ensure a robust, reliable reactor for the generation of H_2_O_2_ that will be subsequently catalyzed to hydroxyl radicals on a heterogeneous Fenton catalyst.

### Heterogeneous Catalyst Material Selection

**FIGURE 4 |:**
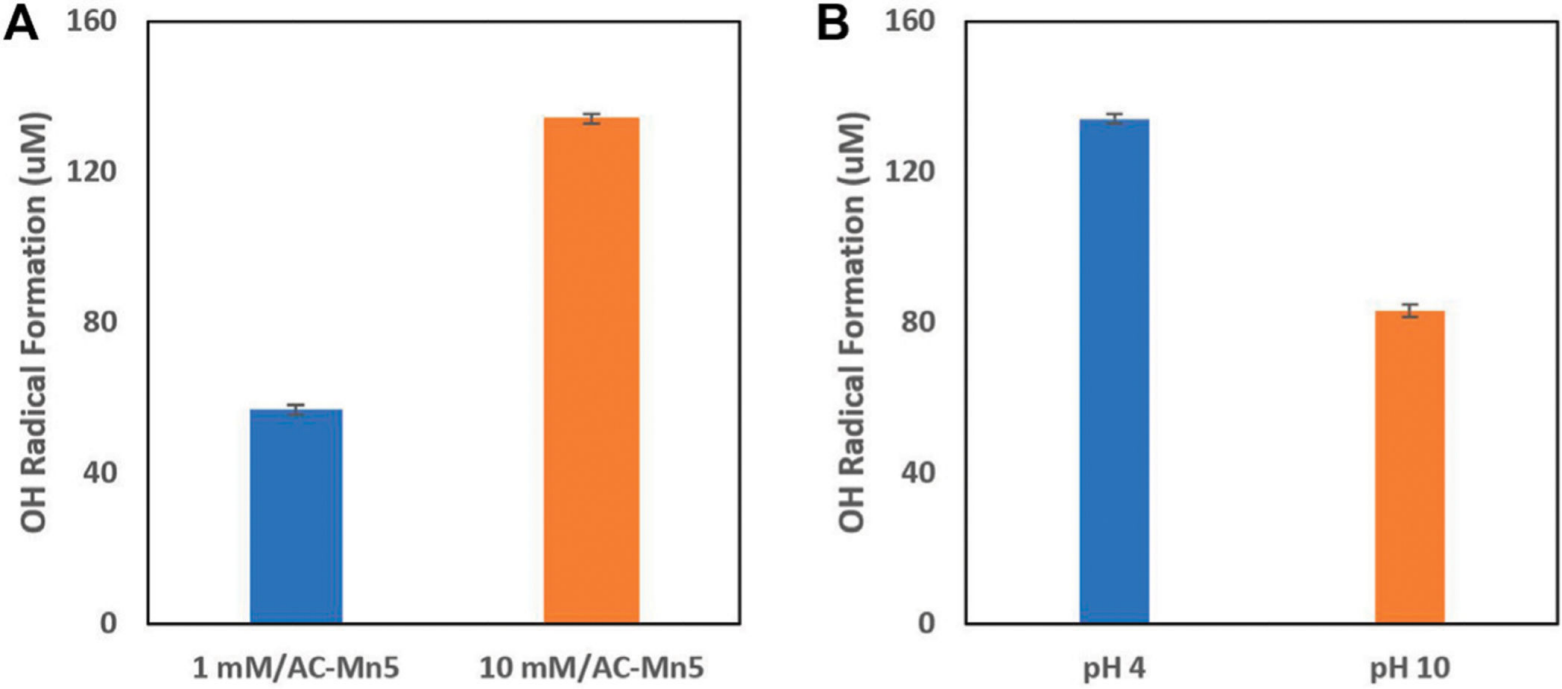
**(A)** H_2_ O_2_ concentration on hydroxyl radical formation and **(B)** Hydroxyl radical formation at varying pH values.

For heterogeneous catalysts, the wide working pH range and reusability/stability of these catalysts is beneficial for implementation in the proposed single flow-through redox reactor. Due to the nature of the different chemical properties of the MCs and IHEs proposed for degradation, a catalyst that can not only function over a wide range of pH values but also be implemented in an engineered solution and remain resilient, robust, and maintainable is vital. The traditional disadvantages that come with heterogenous Fenton catalysts are both the harsh synthetic conditions of catalysts as well as high synthesis costs. These limit the ability for heterogenous catalyst implementation in industrial treatment systems. However, as stated previously, GAC is an inexpensive and innocuous support for a host of catalytic materials and is manipulable for use in many engineering applications. GAC impregnated with manganese dioxide has been shown to produce effective Fenton-like generation of hydroxyl radicals. In previous research that utilized an analogous impregnation technique, the MnO_2_ nanocatalyst showed elimination of mesopore and micropore volume due to filling of these pores with the oxide determined by nitrogen adsorption-desorption isotherms at liquid N_2_ temperature 77 K (Saroyan et al., 2019). Figure 4 shows the generation of hydroxyl radicals with this heterogenous Fenton-like catalyst at different concentrations of influent H_2_O_2_ and initial pH values. It is evident that with increasing H_2_O_2_ concentrations, the formation of hydroxyl radicals increases as well, though 10 mM is a high concentration that is not feasible with these currently applied *in-situ* electro-Fenton generation techniques. However, this is beneficial to show the efficacy of this catalyst to produce heterogenous Fenton-like results. Additionally, although the catalyst is less effective at neutral and alkaline pH values, it is still within an acceptable range of ROS production.

### Batch Removal of RDX by AC-Mn5 Catalyst

**FIGURE 5 |:**
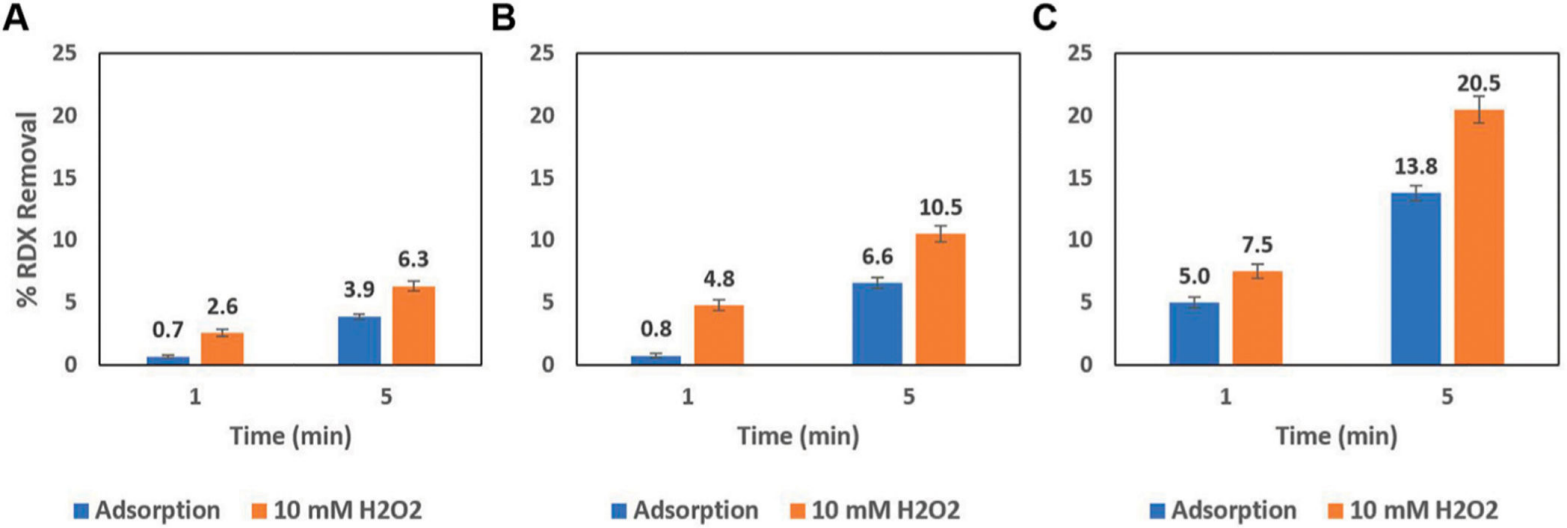
Batch 25 mg/L RDX removal due to **(A)** 10 mg (8 mg AC-Mn5/mg RDX) **(B)** 25 mg (20 mg AC-Mn5/mg RDX), and **(C)** 50 mg (40 mg AC-Mn5/mg RDX) of AC-Mn5 catalyst.

While the results in Figure 4A are the outcome of 100 mg of this AC-Mn5 catalyst in 50 ml of benzoic acid solution, Figure 5 shows the results of varying H_2_O_2_ concentrations and catalyst mass compared to simple adsorption upon the GAC surface. Even with a very small amount of the heterogenous Fenton-like catalyst AC-Mn5, there is increased degradation due to the generation of hydroxyl radicals. Visible in Figure 1, utilizing GAC as a support has the added benefit of adsorption of the pollutant, which is the primary use of activated carbon in traditional physicochemical treatment solutions. Therefore, the manner in which this catalyst is implemented within the proposed single flow-through reactor is of vital importance to ensure Fenton-like reactions are indeed occurring and a significant driver for pollutant removal.

**FIGURE 6 |:**
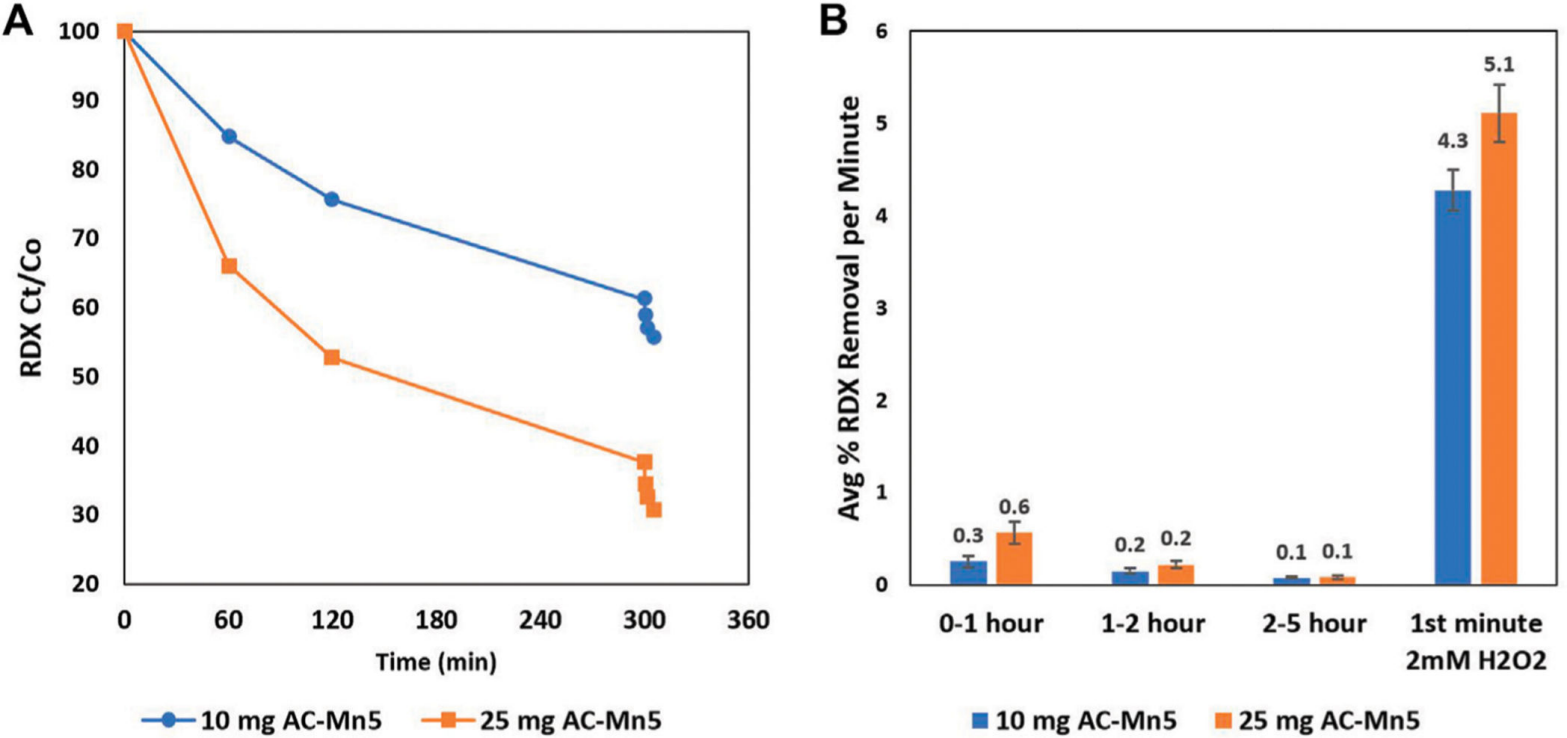
Batch 25 mg/L RDX removal with 10 (8 mg AC-Mn5/mg RDX) and 25 mg (20 mg AC-Mn5/mg RDX) AC-Mn5 catalyst for 5 h adsorption and subsequent 5-min catalysis with 2 mM H_2_O_2_ **(A)** RDX Ct/Co **(B)** Normalized RDX removal per minute.

Figure 6 details batch experiments conducted with minimal AC-Mn5 mass for extensive adsorption followed by catalysis with 2 mM H_2_O_2_. Results indicate that even after a significant portion of the adsorptive capacity of the catalyst is utilized, the AC-Mn5 modification is able to rapidly degrade the RDX compound with are moval rate ∼10X that of the initial adsorptive rate. This shows the removal power of the parent compound RDX by the AC-Mn5 modification long after the adsorptive capabilities of the carbon backbone have been exhausted.

### Combined H_2_O_2_ Electrogeneration and RDX Removal

**FIGURE 7 |:**
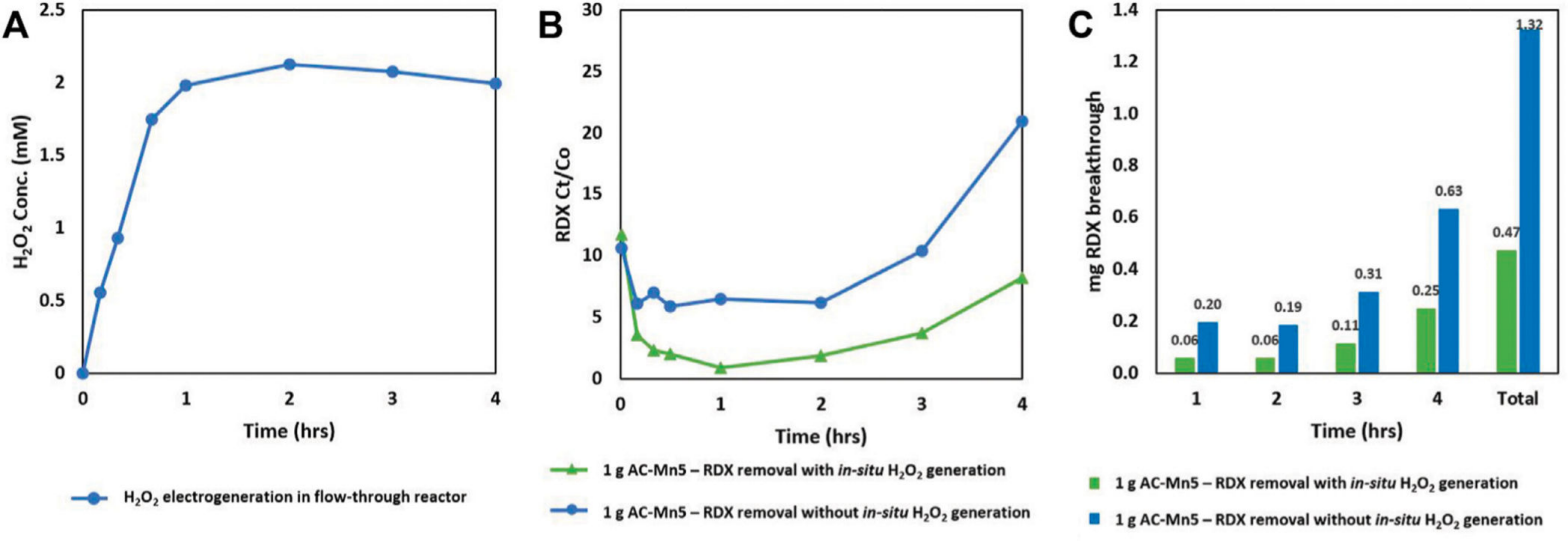
Combined H_2_O_2_ generation and RDX removal by adsorption + H_2_O_2_/AC-Mn5 catalysis under flow **(A)** Sustained H_2_O_2_ electrogeneration with optimized CCMPL cathode **(B)** RDX Ct/Co utilizing 1 gram AC-Mn5 with/without *in-situ* H_2_O_2_ electrogeneration, and **(C)** Mass RDX breakthrough of columns with/without *in-situ* H_2_O_2_ electrogeneration.

Figure 7 is the result of the combined electro-Fenton generation of H_2_O_2_ and catalysis with AC-Mn5 in which 1 Gram of the AC-Mn5 catalyst was placed in a packed bed adsorption column with 25 mg/L of the RDX solution, electrogenerated H_2_O_2_, and electrolyte were dripped through the column. Figure 7B indicates the RDX removal due simply to adsorption onto the catalyst surface as well as the results after first electrogenerating H_2_O_2_ *in-situ* utilizing the CCMPL cathode followed by adsorption/degradation through the packed AC-Mn5 column.

These results indicate that the coupled Fenton optimization of electrogenerated H_2_O_2_ and heterogenous catalyst is more effective in rapidly removing RDX and sustaining a low breakthrough concentration for the duration of the flow test. There is an extremely low breakthrough of 2–3% after 2 h in the coupled Fenton column compared to 6% breakthrough in the adsorption column. However, there is more rapid breakthrough with the adsorption only column, with 10 and 20% breakthrough at hours 3 and 4, respectively. This breakthrough rate is much faster than the 4 and 8% breakthrough at hours 3 and 4, respectively, in the coupled Fenton column. While adsorption accounts for a much more significant portion of the removal, the heterogenous Fenton-like degradation that occurs on the catalyst surface in the presence of *in-situ* generated H_2_O_2_ extends the efficacy of removal and leads to a slower RDX breakthrough rate.

## DISCUSSION

### Optimization of Reactor Parameters to Maximize H_2_O_2_ Electrogeneration

To generate ROSs for degradation of MCs and IHEs, the two Fenton optimization techniques chosen were the heterogenous Fenton and electro-Fenton methods. While each single optimization technique has its own advantages and disadvantages, novel methods for implementing each singly or coupled make them dually advantageous. Electro-Fenton generation of H_2_O_2_ *in-situ* upon a cathode surface is advantageous for the single flow-through reactor proposed. Removing any adjustment to the pollutant influent stream prior to degradation is beneficial from an economical and efficacy standpoint, and electro-Fenton removes the handling, transportation, and storage of H_2_O_2_.

The cathode material choice and orientation optimization of the utilized CCMPL cathode is due primarily to the hydrophobic nature of this material. Previous research has shown that unmodified/hydrophilic carbonaceous materials have low yields for H_2_O_2_ generation per cathode surface area (Qiang et al., 2002; Wang et al., 2005) while carbon cathodes modified with polytetrafluoroethylene (PTFE) produce much higher yields of H_2_O_2_ generation per surface area (Ramirez et al., 2007). This modification of cathode materials to include the fluorocarbon PTFE introduces a hydrophobic layer that sharply reduces cathodic flooding and enables oxygen distribution to increase the efficacy of electrochemical reduction of O_2_ on the cathode surface (Zhou et al., 2007). Whereas optimized PTFE layering can increase surface area by roughly 2.5 times and pore volume by 20 times for O_2_ reduction surface sites, an overabundance of PTFE layers can actually decrease cathodic flooding, oxygen distribution, and H_2_O_2_ generation efficiency (Yu et al., 2015). The CCMPL material utilized in the present work is a woven carbon fiber cloth with a thickness of 0.365 mm and one side treated with PTFE (dubbed the microporous layer). As an oxygen diffusion electrode, this PTFE layer allows for the slower diffusion of the O_2_ generated from the Ti/MMO anode set below the CCMPL cathode through the woven carbon cloth and thus increased generation of H_2_O_2_.

Figure S3 provided in the supplemental shows the ideal shape and cell configuration for the management of gaseous O_2_ bubbles formed on the anode surface. As mentioned previously, the orientation of the CCMPL cathode in which bubble oversaturation becomes inevitable initially causes a rapid increase in the concentration of H_2_O_2_ generated. However, this subsequently leads to a gaseous cavity on the underside of the PTFE coated CCMPL which severely impedes the cathode’s contact with the electrolyte and thus maintaining constant current flow between the anode and cathode. This precipitous drop in voltage leads to the inevitable decrease in H_2_O_2_ electrogeneration. The solution found within this system–labeled in the supplemental as ‘large diameter cathode’ in Supplementary Figure S2–is to orient the flexible CCMPL cathode in such a way as to create a convex shape facing the anode with either small gaps ensured between the cathode edge and reactor wall or small incisions made near the edge to cause O_2_ bypass should oversaturation occur. Supplementary Figure S3 in the supplemental shows a schematic of how bubble coalescing can form oversaturation and the implemented solution to this issue.

The fact that the CCMPL is a cloth material enables easy manipulation of the cathode surface into the desired convex shape to ensure radial sliding and coalescing of O_2_ bubbles in the center of the reactor and maximize diffusion. Either small gaps between the edge of this cathode and reactor wall as well as minor incisions near the edges of the circular cathode will ensure that the coalesced bubbles do not form a cavity that separates the cathode from the electrolyte. Though the incisions and gaps between the cathode and reactor wall may seem to create preferential flow paths that short circuit the cathode and reduce efficiency, the bubble formation and diffusion ensure the reactor is well-mixed and lead to high yield of hydrogen peroxide. This solution was formed with the initial run of experiments and all further optimization for other parameters was conducted with this cathode orientation/manipulation.

The following equations detail the anodic oxidation of water and subsequent cathodic reduction of O_2_ to form H_2_O_2_ (Fukuzumi et al., 2018).


(2) $$2H_{2}O\to O_{2}+4e^{-}+4H^{+}$$

(3) $$O_{2}+2e^{-}+2H^{+}\to H_{2}O_{2}$$

(4) $$H_{2}O_{2}+4H^{+}+4e^{-}\to 2H_{2}O$$

(5) $$O_{2}{\mathrm{+2H}}_{2}O+4e^{-}\to 4{\mathrm{OH}}^{-}$$

The Ti/MMO anode and CCMPL cathode are linked to the same power source with the capability for a variable voltage and constant current flow to these two electrodes. As mentioned previously, the current density applied is the most sensitive to the effective generation of H_2_O_2_ due to O_2_ generation described in Eq. 2, the H_2_O_2_ generation described in Eq. 3 *via* 2-electron reduction, oversaturation as shown in Supplementary Figure S2, and possible parasitic reduction of H_2_O_2_ on the cathode surface described in Eq. 4. First, O_2_ is generated *in-situ* via an oxygen evolution reaction (OER) on the utilized Ti/MMO (IrO_2_ and Ta_2_O_5_ coating on titanium mesh) anode where O_2_ bubble formation is dependent on the applied current to the electrodes (Zhou et al., 2019). This same current is applied to the CCMPL cathode, situated above the anode to allow for O_2_ bubble ascension due to gravity/buoyancy, to initiate the 2-electron oxygen reduction reaction (ORR) shown in Eq. 3.

These two are inextricably linked, as the current that is applied to the anode is identical to that applied at the cathode. *A priori* it may seem that the larger the current applied to the anode for OER, the more oxygen available for ORR on the cathode to produce H_2_O_2_. While one drawback is the rapid bubble formation and oversaturation that occurs with higher applied currents - addressed with the orientation/manipulation of the CCMPL cathode–there is indeed another reaction described in Eq. 4 that describes cathodic H_2_O_2_ decomposition. With the increased flow of electrons at higher currents and the available surface area available for the distribution of these electrons on the 2-dimensional CCMPL cathode, H_2_O_2_ that is generated by ORRs on this surface may be subsequently and rapidly decomposed back to water due to being subjected to additional electron flow at higher currents. The CCMPL cathode is a highly electroactive surface with an increased ability for rate transfer of electrons to the bulk solution under flow conditions, leading to the capability of these 4-electron reduction reactions at increased current densities that may decompose electrogenerated H_2_O_2_. This is evident that even with the reoriented CCMPL cathode in Supplementary Figure S3, the rapid generation of H_2_O_2_ at 200 mA applied current leads to H_2_O_2_ decomposition and a peak yield far below that of 150 mA as seen in Figure 2A. At an applied current of 150 mA–equating to a current density of 9.5 mA/cm^2^–the CCMPL cathode is able to create an optimal environment for cathodic 2-electron ORRs to occur with high H_2_O_2_ yields and minimal decomposition by additional 4-electron reduction reactions.

The flow rate of the electrolyte through the reactor dictates the possible throughput of contaminants and the mass available for degradation within a given timeframe. For the CCMPL orientation optimization as well as the current intensity optimization for H_2_O_2_ yield, a flow rate of around 2 ml/min was utilized. As this flowrate increased, the concentration of H_2_O_2_, in mg/L, was steady for the first 30 min but reached steady state at a lower concentration for each increase in rate. However, because the reactor is under flow conditions, the mass of H_2_O_2_ electrogenerated per minute of operation is a more effective metric to show the effectiveness of the system for the follow-on implementation of a modified-AC column. Thus, Figure 2D shows that with increased flow rate, the mass of H_2_O_2_ generated increases when normalized per minute of operation. With an increased throughput rate, the volume passed through the reactor after 30 min at 2, 5, and 10 ml/min is 60, 150, and 300 ml respectively. Along with this improved mass generation, the current efficiency of the CCMPL cathode increases with increased flowrate. With the flow rates of 2,5, and 10 ml/min, the steady state current efficiency is 5, 11.5, and 23%, respectively. This is due to the higher bulk volume throughput increasing the efficiency of electroperoxidation based on Eq. 1. The fact that the system is able to generate similar concentrations, increased mass flow rates, and greater current efficiencies with increased throughput rates reveals the scalability of such a reactor.

The initial pH of the influent has minor consequence on either the rate of H_2_O_2_ production or the steady state concentration. Figure 2B shows that samples taken at all times during the various pH influent runs were within applicable standard deviations, with variability due simply to CCMPL cathode orientation and bubble saturation inconsistencies. Both H_2_SO_4_ and NaOH were used as acid and base, respectively, to adjust the influent pH values due to their dissolution to Na^+^ and SO_4_^2−^ ions. The electrolyte utilized, Na_2_SO_4_, would not have been affected chemically by the addition of this acid and base. However, the conductivity of the electrolyte in the system may be strengthened/weakened depending on which was utilized, though the low variability in H_2_O_2_ production seems to make these additions inconsequential. The explanation as to why the results are similar across a wide range of influent pH values lies in the pH self-regulating nature of the H_2_O_2_ production system. Supplementary Figure S4 shows the reactor setup and the pH values obtained at separate locations within the system with an acidic and basic influent.

The different column locations 1,2, and 4 refer to the areas before the anode, between the anode and cathode, and after the cathode, respectively. With widely varying influent pH values, the solution after passing the anode reaches a steady pH value in the range of 2.5–3. This tracks with the oxidation of H_2_O shown in Eq. 2 forming both gaseous oxygen and H^+^ ions with any additional OH^−^ ions in the basic influent siphoning H^+^ ions to form the marginal increase in pH at port two in Figure 1. At port 4, following reduction on the cathode to form H_2_O_2_, the acidic regime is changed to basic due to the formation of OH^−^ ions regardless of the pH of the influent due to Eq. 5. This port is situated far above the cathode surface, giving the average pH in the bulk solution leaving the electrochemical reactor. This high pH value is undesirable in traditional Fenton process which utilize Fe^2+^/Fe^3+^ cycling to produce hydroxyl radicals due to the precipitation of ferrihydrite (iron sludge). Because the pH regime following the cathode where the H_2_O_2_ is generated is basic, the use of heterogenous Fenton or Fenton-like catalysts are ideal as they can produce hydroxyl radicals without forming undesirable precipitates.

Determining the longevity of use of the CCMPL cathode is important to understand the feasibility of this system to handle a large volume of wastewater with minimal maintenance as well as cost effectiveness of the CCMPL material. Figure 3A shows the result of nine consecutive H_2_O_2_ production runs with the same CCMPL cathode. resulting in 63% yield in the concentration of H_2_O_2_. As stated above, the maximum effectiveness was maintained after nearly 12 h of operation, which while this was running at ∼2 ml/min does not equate to a large volume of waste, the fact that this material can generate a large mass of H_2_O_2_ per minute of operation makes this more scalable. Figure 3B reveals the CE of the CCMPL cathode utilizing the optimal conditions described in Figure 2 and over the course of nine consecutive tests with an operation time of over 1200 min. While the highest CE obtained was a little over 6%, this maximum efficiency was maintained for six of these consecutive tests, with the CE diminishing to just over 3% with the final trial. While results indicated in previous research have CEs much higher (Wang et al., 2022), these are obtained in batch with superhydrophobic manufactured cathodes utilizing air diffusion. Indeed, these current efficiencies reflect the volume treated after 2 h. As these high steady-state H_2_O_2_ concentrations remain for longer tests, these current efficiencies will increase over the course of the continual, flow-through experiments and with higher throughput flowrates. Figure 3A results are similar to stability tests observed with other hydrophobic carbon cathodes (Yu et al., 2015) and shows that this CCMPL cathode is reasonably stable and reusable in a scaled-up process with minimal maintenance.

### Effectiveness of AC-Mn5 Catalyst in RDX Removal

Activated carbon impregnated with ∼5% w/w MnO_2_ nanopowder is an effective and efficient activated carbon modification utilizing catalytic materials to elicit Fenton-like catalysis. While other catalytic materials such as iron oxychlorides may be more effective in producing higher concentrations of hydroxyl radicals, their complex synthesis and the manner in which they are implemented onto activated carbon backbones are not operationally or economically viable for scale-up. Eqs. 6–8 detail the possible catalysis of H_2_O_2_ in the presence of MnO_x_ to produce hydroxyl radicals and other radical variations (Wenmei Wang et al., 2016).


(6) $${\mathrm{Mn}}^{4+}+H_{2}O_{2}\to H^{+}+{\mathrm{HO}}_{2}.+{\mathrm{Mn}}^{3+}$$

(7) $${\mathrm{Mn}}^{3+}+H_{2}O_{2}\to {\mathrm{OH}}^{-}+\mathrm{OH}.+{\mathrm{Mn}}^{4+}$$

(8) $$H_{2}O_{2}+{\mathrm{HO}}_{2}.\to \mathrm{OH}\text{.}+O_{2^{-}}.+H_{2}O$$

The mechanisms for the production of hydroxyl radicals seen in Eqs. 6–8 bear a resemblance to the traditional Fenton iron cycling with the oxidation and reduction of Mn(IV) and Mn(III), respectively. Figure 4 shows the formation of hydroxyl radicals formed in the presence of varying pH values and H_2_O_2_ concentrations by indirectly measuring the formation of 4-hydroxybenzoic acid during the radical induced hydroxylation of benzoic acid. These hydroxyl radicals, and to an extant the superoxide and hydroperoxyl radicals, are able to non-selectively oxidize pollutants and are especially viable for treatment of recalcitrant persistent organic pollutants. Though RDX is utilized as a model contaminant in degradation experiments, the non-selectivity and high reduction potentials of hydroxyl radicals produced from the Fenton-like catalysis of H_2_O_2_ on the surface of the AC-Mn5 catalyst are viable to oxidize a range and mixture of munitions constituents and insensitive high explosives.

The difficulty in quantifying the efficacy of the catalyst in both hydroxyl radical formation and RDX degradation lies in the simultaneous adsorption onto the carbon surface as well as Fenton-like degradation. While 10 mM of H_2_O_2_ is much higher than can be achieved by the electro-Fenton process utilizing the CCMPL cathode and optimal flow conditions, the high concentration is beneficial to show the immediate oxidation of RDX in addition to rapid adsorption onto the carbon surface that occurs. Figure 5 details three increasing masses of AC-Mn5 catalyst with and without 10 mM H_2_O_2_, with the values of 10, 25, and 50 mg being light but adequate masses for these 50 ml batch experiments due to the low RDX concentration of 25 mg/L. With the 10 mg AC-Mn5 experiments, there was more rapid removal of RDX due to degradation in the first minute in the presence of H_2_O_2_, 2.6% degradation/adsorption versus 0.7% adsorption, compared to removal from 1 to 5 min, 3.75% additional degradation/adsorption versus 3.25% additional adsorption. This is similar to the 25 mg AC-Mn5 batch experiment, with 4.8% degradation/adsorption versus 0.75% adsorption in the first minute and 5.7% additional degradation/adsorption compared to 5.8% additional adsorption from 1 to 5 min. However, a doubling of this mass and subsequently w/w ratio of mg AC-Mn5/mg RDX leads to 7.5% degradation/adsorption versus 5% adsorption in the first minute and 13% additional degradation/adsorption compared to 8.8% additional adsorption from 1 to 5 min.

The lower mass of catalyst in the RDX solution equates to a lower number of active sites for adsorption and catalysis and may suggest that once sites are utilized for either adsorption or catalysis, degradation will be minimal. Although H_2_O_2_ has the ability to ‘age’ the AC-Mn5 catalyst, the concentrations produced in electroperoxidation are inconsequential to produce a drop in catalytic potential. Figure 6 shows that after 5 h of adsorption onto 10 and 25 mg of catalyst (8 and 20 mg AC-Mn5/mg RDX w/w ratio), there is significant degradation due to only 2 mM H_2_O_2_. Adsorptive sites are significantly exhausted prior to the addition of external H_2_O_2_ for the 25 mg catalyst, with an average of 0.5, 0.2, and 0.08% RDX removal by adsorption per minute for hours 1, 2, and three to five, respectively. The addition of the external H_2_O_2_ leads to greater than 5% removal of the RDX parent compound during 1 min of contact time. While the removal is not as substantial, this trend and subsequent degradation efficiency after adsorptive capacity is nearly reached is similar in the 10 mg catalyst experiment. These results lead to two distinct conclusions, with one being the efficacy of the AC modification after the carbon support is saturated and the manner in which this catalyst should be implemented in an engineered solution. While the different masses of catalysts utilized for Figure 5 show the impact of a higher number of adsorptive and catalytic sites for RDX removal, the rapid rate of removal due to RDX degradation by Fenton-like oxidation is promising for implementation in a packed bed adsorption column in which the model pollutant percolates through the column with probability for catalysis increasing with movement through the column.

### Coupled Fenton Optimization Techniques in Flow-Through Reactors for RDX Removal

The prior electrogeneration of H_2_O_2_ utilizing the electroperoxidation aspect of the electro-Fenton technique with subsequent adsorption and degradation in the packed bed AC-Mn5 column and the benefits thereof are clear in Figure 7. For these runs, due to the low concentration of RDX, only 1 Gram (4 cm^3^ within the column) of the AC-Mn5 catalyst was utilized in order to show the disparity in breakthrough percentages between the adsorption column and dual adsorption/catalytic degradation column. Full removal of the pollutant RDX was achieved with a column filled with 5 grams (20 cm^3^ within the column) AC-Mn5 but the effectiveness of the electrogenerated H_2_O_2_ as well as the AC-Mn5 modification techniques over simple adsorption was not evident. Therefore, 1 Gram of the AC-Mn5 catalyst along a length of 1.5 cm in the column was utilized and the insights made more evident as to the added benefit of MnO_2_ impregnation and electrogenerated H_2_O_2_ for RDX removal.

While it is evident that adsorption onto the carbon surface is the driving factor for significant removal of the parent compound RDX, there are a number of things that make the addition of *in-situ* generated H_2_O_2_ superior to removal of the contaminant by adsorption. First, the rapid adsorption that occurs in the column is complemented by additional degradation of the compound via hydroxyl radical induced oxidation. This leads to 98% removal rate within the first 10 min of operation when the steady-state concentration of hydrogen peroxide has not reached its maximum. After 1 and 2 h of operation, as the steady-state concentration of H_2_O_2_ production reaches its steady-state maximum of >2 mM, the heterogenous Fenton-like oxidation accounts for only 5% added removal of the parent compound RDX as adsorption remains the dominant removal pathway. However, as the column becomes saturated, the rate of breakthrough due to adsorption increases rapidly while degradation still occurs. In this case, while adsorptive capacity does decrease with time in the column including electrogenerated H_2_O_2_, the rate of RDX breakthrough is much slower due to the added degradation pathway and more of the parent RDX compound can be treated effectively with the same mass. This not only extends the longevity of the AC-Mn5 catalyst as a removal mechanism, but in fact degrades the RDX to smaller organic byproducts. Indeed, Figure 7C shows that there is three times as much RDX mass that is allowed to break through the column and reach the effluent if catalysis by electrogenerated H_2_O_2_ is not utilized. This decrease in breakthrough mass by the combined columns due to only 1 Gram of the AC-Mn5 catalyst would be exponentially greater with a larger mass/volume and continued throughput of the pollutant.

To understand the intermediate and follow-on byproducts of RDX oxidation by powerful oxidants, it is important to understand the efficacy of RDX oxidation as well as certain degradation mechanisms. Due to the chemical nature of the RDX molecule, its heterocyclic ring structure and nitro groups are resilient and not easily oxidized by hydroxyl radicals (Chen et al., 2008). This reveals RDX’s low reactivity to hydroxyl radical oxidation from a kinetics standpoint, but the fact remains that with the right reactor setup significant removal by oxidation from hydroxyl radicals may occur. This is supported by research that shows that greater steady-state concentrations of hydroxyl radicals in contact with RDX leads to faster degradation rates (Bose et al., 1998a). It is suggested that the nitro groups attached to the RDX ring structure as well as N and C within the ring structure are oxidized by hydroxyl radicals to nitrate and organic by-products, respectively.

The organic byproduct observed via the analytical techniques described in the experimental was methylenedinitramine (MDNA), otherwise known as Medina, and has been detected in previous electrocatalytic systems (Wani et al., 2006; Chen et al., 2011). MDNA, a ring cleavage byproduct seen previously following the reaction of RDX with anaerobic sludge (Fournier et al., 2004), is potentially formed by varying degradation pathways, with one course due to direct ring cleavage of the parent RDX compound. Of the inorganic byproducts detected, ammonium (detected by a Fisher ammonia probe), nitrate, and nitrite detail N molecules removed from RDX by oxidation. Detection of ammonium and nitrite in other oxidative systems (Hundal et al., 1997; Bose et al., 1998b) indicate further cleavage, this time of the single N bonds present in the parent RDX compound. Finally, formate and acetate, previously observed byproducts of RDX degradation via alkaline hydrolysis (Gent et al., 2009), detail some of the reactions of the carbon from the RDX compound. While it is safe to say that cleavage of atoms attached to the heterocyclic ring as well as cleavage of the heterocyclic ring itself occurs in this system, the full mineralization of RDX was not detectable via this methodology. However, TOC measurements were conducted as described in the experimental on effluent collected after 1 h of flow through the AC-Mn5 column. The TOC concentration measured was 1 mg/L, which due to the fact that RDX has three carbons within its heterocyclic ring, leads to around a 75% reduction in TOC with this method utilizing 1 Gram of the AC-Mn5 catalyst with electrogenerated H_2_O_2_. Additionally, due to the fact that large concentrations of manganese can be toxic, possible leaching of Mn from the AC-Mn5 column during operation was checked by inductively coupled plasma–mass spectrometry. Samples were collected from the column effluent, acidified with 1% nitric acid to dissolve Mn, and filtered (0.2 micron) to remove any AC particles. Leached Mn concentrations were near 2 mg/L, due to excess MnO_2_ residuals leaving the carbon surface an into the aqueous effluent. In practice, methods will be needed to retain AC-Mn5 particles within the column, perhaps by straining with smaller porous media supports (e.g., sand) or use of replaceable glass filters.

These byproduct findings lead to a multitude of removal mechanisms and parasitic reactions taking place within the packed AC-Mn5 bed reactor in the presence of H_2_O_2_. As the RDX and H_2_O_2_ solution enters the column, rapid adsorption of the RDX onto the activated carbon surface as well as catalysis of H_2_O_2_ via Eqs. 6–8 occurs. This produces the above stated radicals, mainly hydroxyl radicals, which then oxidize the nitro groups and nitrogen/carbon bonds within the ring structure to form formamide, urea, and possibly N-hydroxy formamide and nitroformaldehyde. These by-products may subsequently be either adsorbed or oxidized by hydroxyl radicals further down the column, which is simultaneously adsorbing RDX, catalyzing H_2_O_2_, and oxidizing the parent RDX compound. Finally, the solution exits the column where it is collected for analysis in the HPLC. Due to the lack of retention of polar compounds in the HPLC column, only the RDX removal can be measured. Therefore, tailoring not only the w/w ratio of AC-Mn5 to RDX but the retention time (contact time) of the RDX/H_2_O_2_ solution in the packed bed is key. As shown in Figures 5, 6, the most rapid removal of RDX due to hydroxyl radical oxidation occurs after 1 min, with further oxidation occurring up to 5 min. After this period adsorption is the dominant removal method. In addition, the effectiveness of the AC-Mn5 catalyst in generating hydroxyl radicals is reduced in a basic environment. As shown in Supplementary Figure S4 available in the supplemental and eq. 5, the bulk solution of RDX, electrolyte, and electrogenerated H_2_O_2_ entering the packed AC-Mn5 has a pH value of ∼10. This makes the catalysis of H_2_O_2_ and generation of hydroxyl radicals more reduced than if the bulk solution entering the column was acidic. However, precipitation of iron that typically limits the use of homogenous Fenton catalysts is nonexistent in this setup, allowing for movement towards practical applications. Realizing the full potential of the coupled electro-Fenton/heterogenous Fenton optimization techniques by designing a packed AC-Mn5 bed reactor that maximizes removal by degradation is a key next step in the implementation of the technology to more large-scale applications.

### Conclusion and Future Direction of Research

Mixtures of new IHEs and traditional MCs pose challenges to traditional physicochemical water and wastewater treatment processes due to their persistent and recalcitrant nature. Two optimization techniques to the Fenton process to overcome limitations are electro-Fenton and heterogeneous Fenton. In this present study, the electroperoxidation (cathodic reduction of O_2_ on a cathode) portion of the electro-Fenton technique as well as the use of a heterogenous Fenton-like catalyst for the reduction of H_2_O_2_ to form hydroxyl radicals and oxidize the model pollutant RDX were investigated.

A commercially available hydrophobic carbon cloth cathode with microporous layers was found to be an effective material for the electrosynthesis of H_2_O_2_. A current density of 9.5 mA/cm^2^ applied to the CCMPL cathode results in the highest and most reliable steady state concentration of electrogenerated H_2_O_2_, with this material and design operating well at varying influent pH values as well as with increasing flow rates.

Activated carbon impregnated with ∼5% w/w MnO_2_ nanopowder was determined to be an effective and efficient activated carbon modification to produce heterogenous Fenton-like catalysis of H_2_O_2_. With increasing H_2_O_2_ concentrations and at acidic pH values, the AC-Mn5 catalyst performs exceedingly well at producing hydroxyl radicals. Utilizing activated carbon as a support for the catalytic MnO_2_ nanopowder has the added benefit of adsorption of pollutants, though this study shows that even with an extremely small mass of catalyst the Fenton-like oxidation of RDX is rapid. While adsorption is the dominant removal pathway for RDX with this catalyst, even after significant adsorption nearly to the point of saturation, the AC-Mn5 modification results in significant removal in the presence of H_2_O_2_.

Combining these two techniques into a system that comprises a plug flow reactor for electroperoxidation on the CCMPL cathode followed by removal in a packed AC-Mn5 bed reactor is effective in not only removal of the RDX compound but extended degradation of the recalcitrant pollutant to potential organic by-products and possibly full mineralization. While adsorption is a traditional form of pollutant removal utilized in commercial and industrial water treatment, it simply removes pollutants from one bulk phase to another while keeping the chemical structure of the pollutant intact. In this method, while there is significant adsorption, the benefits of degradation of the RDX compound oxidizes the persistent pollutant so that less is retained in the bulk adsorption phase and the effective longevity of the packed AC-Mn5 bed reactor is extended. Due to the rapid oxidation that occurs on the catalyst surface, the next optimization to occur will be the mass and retention time of the pollutant in the column for optimal degradation and removal. While RDX was used as the model pollutant, this method can be extended to analogous MCs as well as the more persistent IHEs that are emerging as a class of pollutants. Recent literature into the electrochemical regeneration of saturated activated carbon reveals a promising future for the extended operability and economic feasibility of this technology, in that electrochemical systems improve the reusability of sorption materials made from carbonaceous precursors (Santos et al., 2022). By implementing the AC-Mn5 heterogenous catalyst in a system such as this, the lifetime and effective use of these materials may be extended. Additionally, further optimization of this design and CCMPL cathode implementation is needed to maximize the current efficiency of this system as it relates to electricity consumed for optimal H_2_O_2_ yield and total electricity applied. Furthermore, to test the validity of this technique for largescale implementation for a mixture of MCs and IHEs, more complete byproduct determination, degradation pathways, and continuous TOC characterizations of effluent to indicate full mineralization of these compounds is required. Finally, determining the AC-Mn5 reusability and potential release of manganese from this heterogeneous catalyst into the effluent is key for utilizing this as a viable pollutant removal technique.

While current research into other coupled optimization techniques, such as photoelectron-Fenton and sonoelectro-Fenton, is ongoing, their low degradation efficiency and prohibitive cost are less attractive than the presented method. This method, although requiring further optimization in degradation kinetics, reactor design, and prior membrane filtration to ensure solids-free waste streams, is effective in not only removing these pollutants from waste streams but extending the effectiveness of industrial water treatment systems while incorporating an economy of materials and remaining resilient, robust, and maintainable.

## Supplementary Material

Supp. Material

## Data Availability

The raw data supporting the conclusion of this article will be made available by the authors, without undue reservation.
