# Supplementary material for: Heterogeneous Fenton-Like Catalysis of Electrogenerated H2O2 for Dissolved RDX Removal
Source: Front Chem Eng. Author manuscript; Available in PMC 2023 May 17. (PMC10191351; doi:10.3389/fceng.2022.864816)
Supplement: Supp. Material [file NIHMS1840351-supplement-Supp__Material.pdf]

Appendix

Heterogeneous Fenton-like Catalysis of Electrogenerated H_2O_2 for Dissolved RDX Removal

Patrick Compton[1]*, Nazli Rafei Dehkordi[1], Michael Knapp[1], Loretta A. Fernandez[1], Akram N. Alshawabkeh[1], and Philip Larese-Casanova[1]

[1]Department of Civil and Environmental Engineering, Northeastern University, Boston, MA, USA

*Corresponding Author
Patrick Compton
Department of Civil & Environmental Engineering
Northeastern University
360 Huntington Ave, 501 ST, Boston, Massachusetts, 02115, USA
Phone: +1-617-373-4153
Compton.p@northeastern.edu

Keywords: Explosives$_1$, oxidation processes$_2$, heterogenous catalysts$_3$, electroperoxidation$_4$, RDX$_5$

This Appendix contains four figures on materials used as cathodes, cathode orientation within the electroperoxidation column, and the pH regime within this column.

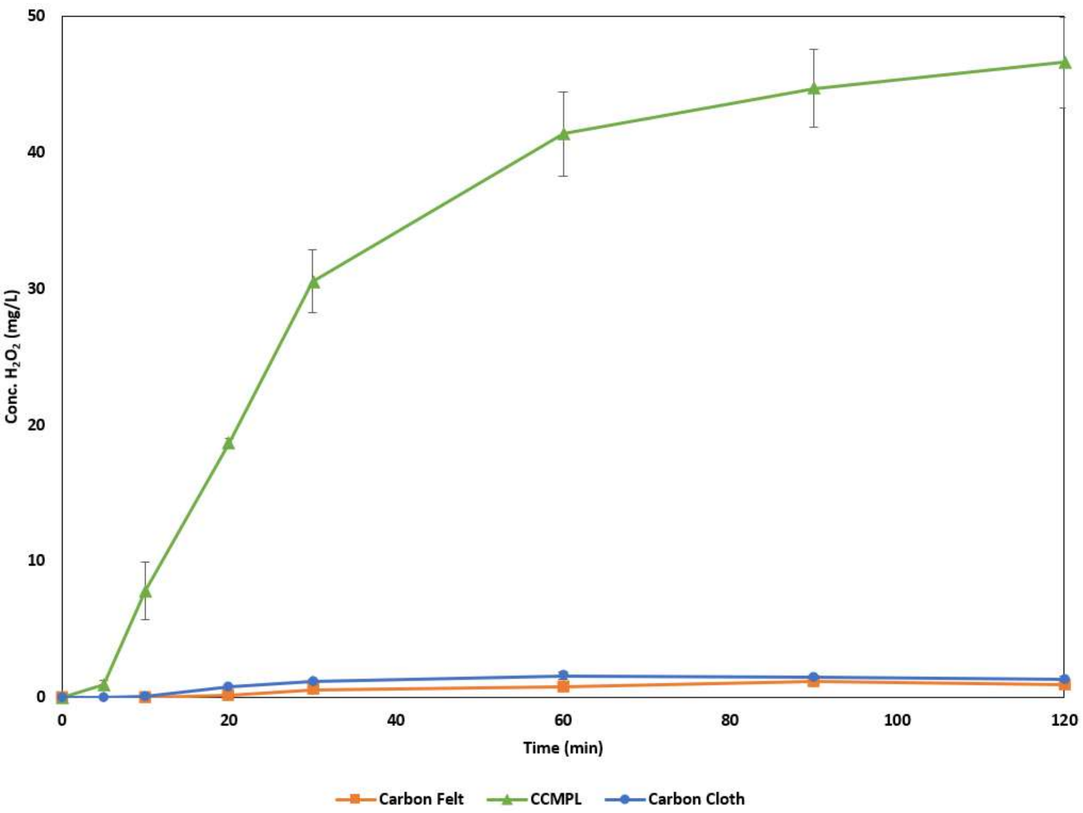

Figure S1. Comparison of hydrophobic/hydrophilic commercially available cathode materials in electrogenerating H_2O_2 in flow-through reactor at 9.5 mA/cm^2 current density

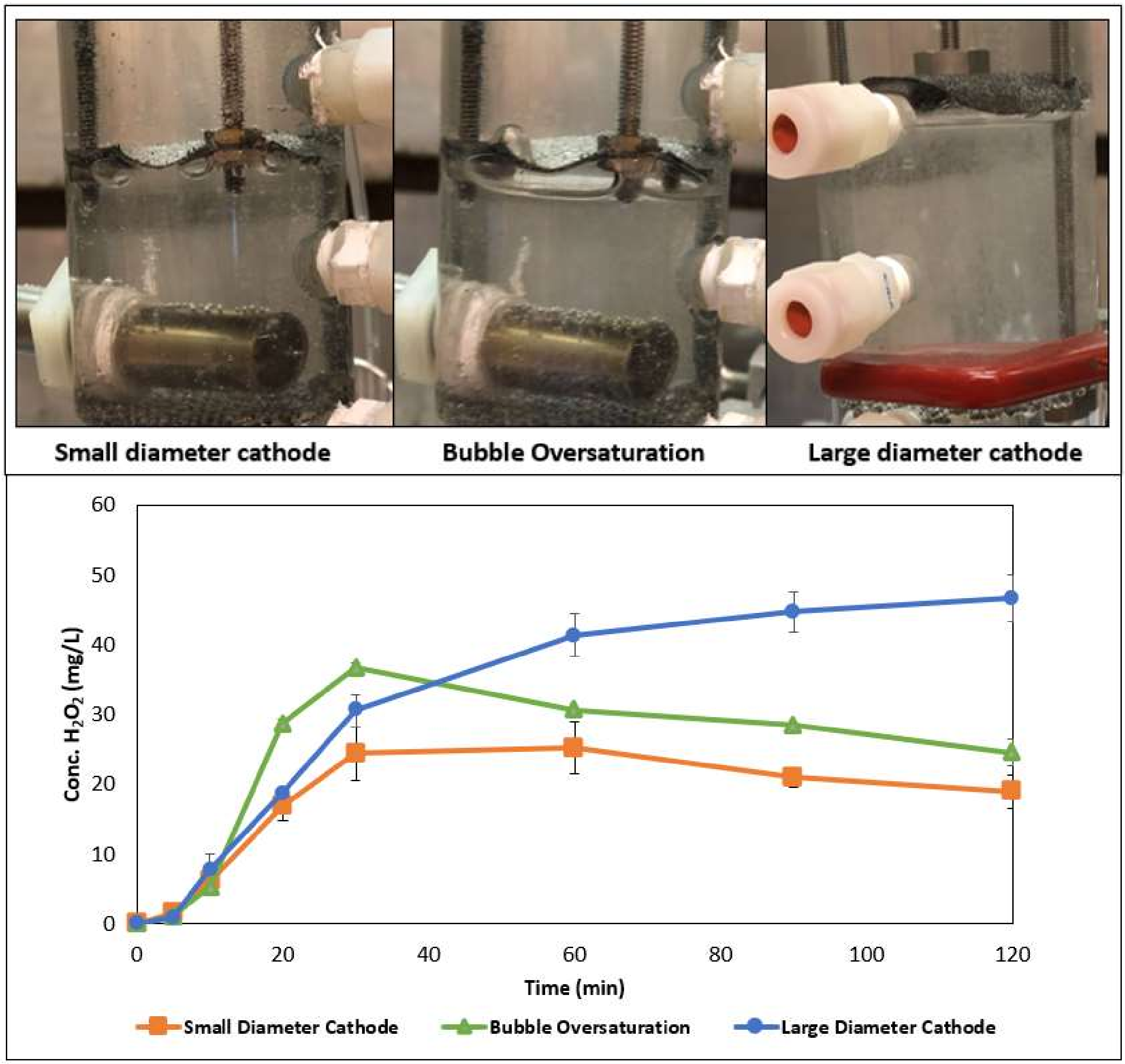

Figure S2. CCMPL cathode size comparison and H_2O_2 generation due to constant O_2 bypass, oversaturation, and designed O_2 bypass

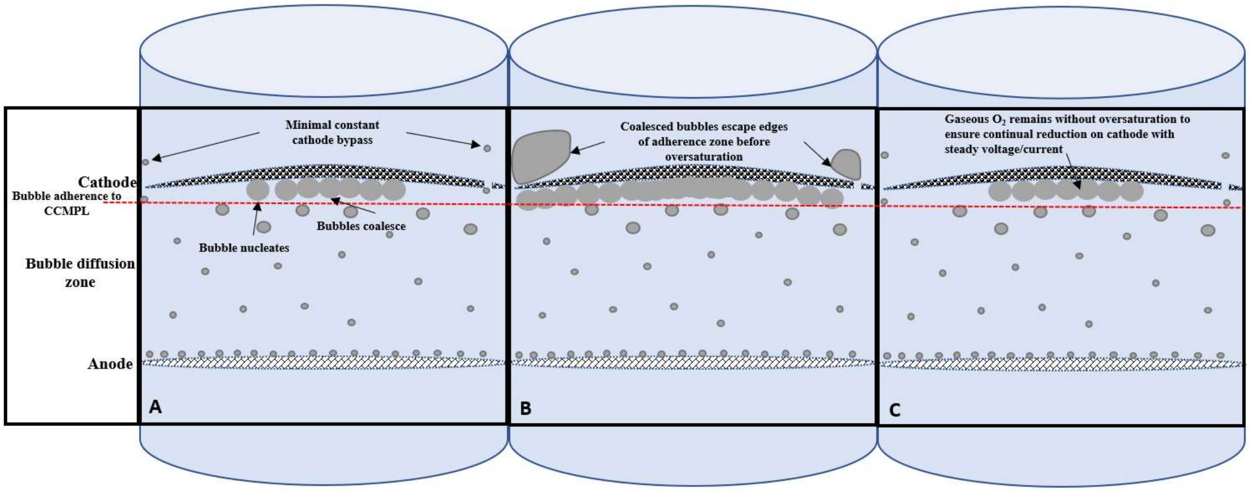

Figure S3. Convex CCMPL cathode with (A) O_2 reduction on PTFE surface, (B) engineered bubble bypass to prevent oversaturation, and (C) continual gas diffusion while ensuring steady voltage/current

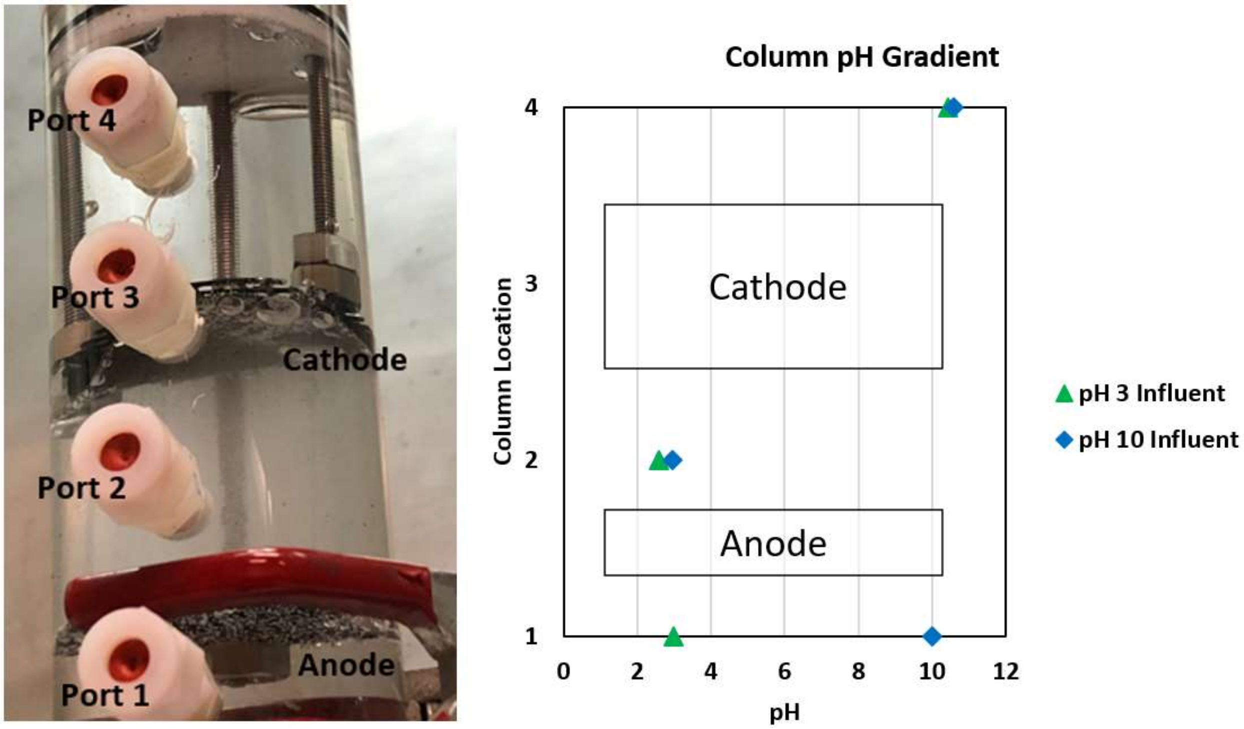

Figure S4. pH self-regulation within electrochemical system
